# Osteogenesis imperfecta mutations in plastin 3 lead to impaired calcium regulation of actin bundling

**DOI:** 10.1038/s41413-020-0095-2

**Published:** 2020-05-22

**Authors:** Christopher L. Schwebach, Elena Kudryashova, Weili Zheng, Matthew Orchard, Harper Smith, Lucas A. Runyan, Edward H. Egelman, Dmitri S. Kudryashov

**Affiliations:** 1 Department of Chemistry and Biochemistry, The Ohio State University, Columbus, OH 43210 USA; 2 Molecular Cellular and Developmental Biology graduate program, The Ohio State University, Columbus, OH 43210 USA; 3 Department of Biochemistry and Molecular Genetics, University of Virginia, Charlottesville, VA 22908 USA; 4 Biophysics graduate program, The Ohio State University, Columbus, OH 43210 USA

**Keywords:** Bone, Pathogenesis

## Abstract

Mutations in actin-bundling protein plastin 3 (PLS3) emerged as a cause of congenital osteoporosis, but neither the role of PLS3 in bone development nor the mechanisms underlying PLS3-dependent osteoporosis are understood. Of the over 20 identified osteoporosis-linked PLS3 mutations, we investigated all five that are expected to produce full-length protein. One of the mutations distorted an actin-binding loop in the second actin-binding domain of PLS3 and abolished F-actin bundling as revealed by cryo-EM reconstruction and protein interaction assays. Surprisingly, the remaining four mutants fully retained F-actin bundling ability. However, they displayed defects in Ca^2+^ sensitivity: two of the mutants lost the ability to be inhibited by Ca^2+^, while the other two became hypersensitive to Ca^2+^. Each group of the mutants with similar biochemical properties showed highly characteristic cellular behavior. Wild-type PLS3 was distributed between lamellipodia and focal adhesions. In striking contrast, the Ca^2+^-hyposensitive mutants were not found at the leading edge but localized exclusively at focal adhesions/stress fibers, which displayed reinforced morphology. Consistently, the Ca^2+^-hypersensitive PLS3 mutants were restricted to lamellipodia, while chelation of Ca^2+^ caused their redistribution to focal adhesions. Finally, the bundling-deficient mutant failed to co-localize with any F-actin structures in cells despite a preserved F-actin binding through a non-mutation-bearing actin-binding domain. Our findings revealed that severe osteoporosis can be caused by a mutational disruption of the Ca^2+^-controlled PLS3’s cycling between adhesion complexes and the leading edge. Integration of the structural, biochemical, and cell biology insights enabled us to propose a molecular mechanism of plastin activity regulation by Ca^2+^.

## Introduction

Osteoporosis is a disease defined by low bone density and disruption of the bone architecture resulting in fragility and fractures.^1^ Hereditary forms of bone fragility called osteogenesis imperfecta (OI) or “brittle bone disease” are mostly linked to dysregulation of Type I collagen.^2^ Approximately 90% of OI cases stem from mutations in collagen I genes,^3,4^ while most of the remaining forms affect collagen-processing enzymes involved in collagen folding, posttranslational modifications, intracellular transport, or matrix incorporation.^4^ Recently, several cases of OI with classical clinical manifestations in hemizygous men and a variable phenotype in heterozygous women, but without an obvious link to collagen, were attributed to mutations in an X-chromosome gene coding an actin-bundling protein plastin 3 (PLS3).^5–17^

Among three vertebrate tissue-specific plastin isoforms,^18^ PLS3 (also known as T-plastin) is ubiquitously expressed in solid tissues^19^ and involved in cell migration,^20^ endocytosis,^21^ DNA repair,^22^ and membrane trafficking.^23^ In agreement with the essential role of PLS3 in bone and connective tissue development in vertebrates, a *pls3* knockdown in zebrafish results in craniofacial dysplasia and malformations of body axis and tail,^13^ whereas PLS3 knockout mouse models showed impaired cortical bone acquisition with decreased osteoblast mineralization capacity^24^ and defects in the development of the epidermal basal membrane.^25^ In humans, PLS3 mutations were also associated with a diaphragmatic hernia.^26^ However, a detailed understanding of PLS3’s contribution to any of the above-mentioned cellular processes or to osteogenesis is missing.

**Fig. 1:**
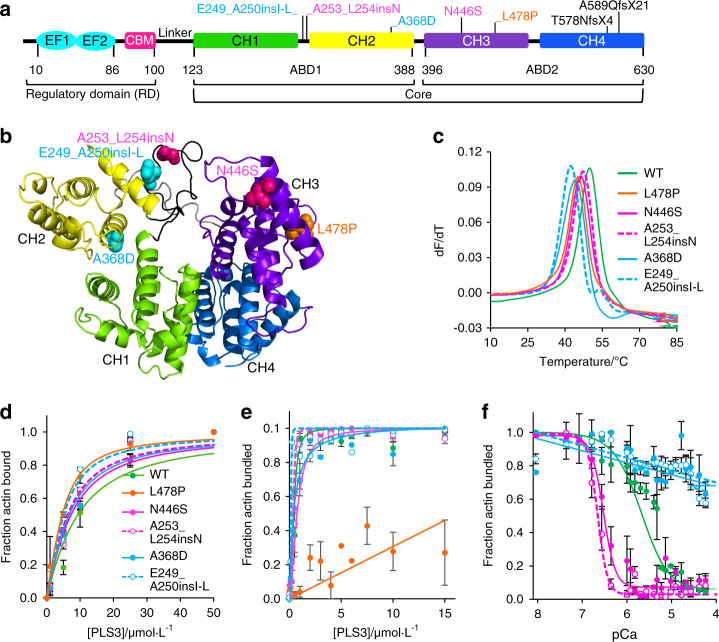
PLS3 domain structure and effects of OI-linked PLS3 mutations on PLS3 properties (see also Supplementary Fig. S1). **a** A schematic diagram of plastin domain structure: EF EF-hands motifs, CBM calmodulin-binding motif, RD N-terminal regulatory domain, CH calponin-homology domains, ABD actin-binding domains, Core actin-binding core domain, Linker a flexible linker separating the CBM and ABD1. PLS3 amino acid residue numbers and the OI-causative PLS3 mutations are shown below and above the diagram, respectively. **b** A homology-based model of the PLS3 actin-binding core (color scheme as in **a**) generated by Phyre2.^86^ **c** Melting profiles of PLS3 osteoporosis mutants were recorded by DSF in three independent repetitions; averaged data were plotted as the negative first derivatives of fluorescence signals versus temperature with transition temperatures (T_m_) summarized in Table 1. F-actin binding (**d**) and bundling (**e**) by WT and mutated PLS3 were analyzed by high- and low-speed co-sedimentation and characterized by K_d_ and [PLS3]_50%_ respectively (given in Table 1). Error bars represent standard errors of the mean of three and two independent repetitions for binding and bundling experiments, respectively (see also Supplementary Fig. S4). **f** Sensitivity of the PLS3 mutants to Ca^2+^ was measured by the decrease in light scattering reflecting the dissociation of plastin-mediated F-actin bundles upon titration with CaCl_2_. Error bars represent standard errors of the mean of three independent experiments. The data were fit to a logistic curve to determine the pCa_50%_ (provided in Table 1)

The domain structure of PLS3 (Fig. 1a) encompasses the N-terminal Ca^2+^-binding regulatory domain (RD) and a core consisting of two actin-binding domains (ABD1 and ABD2). RD contains two EF-hands and a calmodulin-binding motif (CBM), whereas each ABD is assembled from two tandem calponin-homology (t-CH) domains. Binding of Ca^2+^ ions by EF-hands potently inhibits F-actin bundling, but has only a marginal effect on F-actin binding by all human isoforms,^27^ suggesting that only one of the ABD’s binding to actin is inhibited. RD is connected to the ABD core via a linker (Fig. 1a), whose length and likely flexibility precluded, thus far, mapping of the RD’s place in the tertiary structure of plastins; hence, the mechanism of the Ca^2+^-dependent regulation remains unknown.

Of over 20 OI-linked mutations of PLS3 identified to date in osteoporosis patients,^5–17^ five are insertions or missense mutations that are predicted to produce full-length protein, while the remaining are either gene deletions or nonsense/frameshift mutations expected, and in some cases demonstrated,^13,16^ to produce no protein owing to a nonsense-mediated mRNA decay.^28,29^ Of the former five (Fig. 1a, b), a 12-amino-acid insertion E249_A250insIMGHSHSGSCLL^13^ (henceforth designated as E249_A250insI-L) and a single-residue insertion A253_L254insN^13^ are located in ABD1 in the same CH1–CH2 connecting loop, which is disordered in both existing crystal structures of ABD cores of yeast and plant fimbrins.^30^ The remaining three missense mutations are located in the CH2 of ABD1 (A368D),^12^ in a proline-rich loop of CH3 (N446S),^5^ and another loop of CH3 in proximity to a tentative actin-binding surface^31^ (L478P).^14^

In this study, we characterized all five known insertion and missense OI-linked PLS3 mutations leading to a full-length PLS3 protein production, as well as two frameshift mutations (T578NfsX4^9^ and A589QfsX21^6^), which, due to proximity to the 3′-untranslated region, may escape the nonsense-mediated mRNA decay^28,29^ and result in truncated PLS3 variants (Fig. 1a). We provided molecular and cellular insights into the pathological effects of the PLS3 mutants. This analysis allowed classification of the mutations into four groups based on the resulting functional impairment: (1) PLS3 protein loss/instability (gene deletions or nonsense/frameshift mutations), (2) actin bundling-deficient (L478P), (3) Ca^2+^-insensitive (E249_A250insI-L and A368D), and (4) Ca^2+^-hypersensitive (A253_L254insN and N446S). The data imply that the Ca^2+^-regulated F-actin bundling function of PLS3 is essential for bone formation. Despite the well-recognized role of Ca^2+^ in the regulation of vertebrate plastins in vitro, this is the first study, to our knowledge, showing that the fine-tuned Ca^2+^ regulation is crucial for cellular localization and functionality of these proteins. Importantly, location of the mutations perturbing Ca^2+^ sensitivity in both ABDs of the plastin core allowed us to map the position of the RD and propose a mechanistic explanation for the Ca^2+^-dependent inhibition of the plastin/fimbrin family of proteins.

## Results

### Protein instability is a likely reason of OI phenotype in case of the frameshift but not the insertion and missense PLS3 mutations

To check whether the OI phenotype can be a consequence of destabilized/denatured protein, seven recombinant OI PLS3 mutants and wild-type (WT) PLS3 (Fig. 1a, b) were expressed and purified from *Escherichia coli*. While the WT and five missense/insertion PLS3 mutants were soluble, the two truncated variants carrying T578NfsX4 and A589QfsX21 frameshift mutations were largely insoluble (Supplementary Fig. S1a–c). Furthermore, attempts to purify the truncation constructs from the minor soluble fraction failed due to their proteolytic degradation (Supplementary Fig. S1d). Thermal denaturation assessed by differential scanning fluorimetry (DSF) revealed that all seven mutations destabilized the protein to varying degrees, albeit the strongest destabilization was observed for the frameshift mutations (Supplementary Fig. S1e) further substantiating their disruptive nature. The five missense/insertion mutations resulted in moderate protein destabilization (2.6 °C–7.6 °C, Fig. 1c, Table 1), but the transition point did not drop below 42 °C (i.e., was well above the physiological temperature), and no substantial increase in protein aggregation/degradation was observed. Accordingly, all five missense/insertion mutants expressed in human U2OS osteosarcoma cells were detected as a single full-length protein band on a western blot (Supplementary Fig. S7). Therefore, protein destabilization is a likely reason for the disease phenotype by the frameshift but not the missense/insertion mutations. Hence, we focused on characterization of two PLS3 insertion mutants (E249_A250insI-L and A253_L254insN) and three missense mutants (A368D, N446S, and L478P).

**Table 1:** Biochemical properties of PLS3 variants carrying OI-linked mutations

PLS3 construct	T_m_ /°C ± SE	K_d_/μmol·L^−1^ ± SD	[PLS3]_50%_/μmol·L^−1^ ± SD		pCa_50%_ ± SD
			EGTA	Ca^2+^	
Wild type	49.47 ± 0.72	7.41 ± 0.10	0.49 ± 0.28	2.97 ± 1.48	5.46 ± 0.14
L478P	44.93 ± 0.16	2.16 ± 0.36	n/a	n/a	n/a
N446S	46.87 ± 0.07	4.79 ± 0.56	0.58 ± 0.14	6.36 ± 2.46	6.44 ± 0.21
A253_L254insN	45.94 ± 0.95	3.77 ± 0.19	0.53 ± 0.16	2.09 ± 1.55	6.66 ± 0.17
A368D	43.38 ± 0.04	4.22 ± 0.81	0.47 ± 0.15	0.59 ± 0.09	n/a
E249_A250insI-L	41.93 ± 0.18	2.56 ± 0.46	0.10 ± 0.05	0.03 ± 0.04	n/a

*T*_*m*_ melting temperature determined by DSF, *K*_*d*_ equilibrium dissociation constant of PLS3 binding to F-actin, [*PLS3*]_50%_ bundling efficiency of PLS3 (concentration of PLS3 at 50% of F-actin bundled), pCa_50%_ Ca^2+^ sensitivity of PLS3 (-log[Ca^2+^] at 50% reduction in light scattering of actin bundles), *SE* standard error of the mean, *SD* standard deviation of the mean

### L478P mutation abolishes F-actin bundling by PLS3

F-actin binding and bundling abilities of PLS3 mutants were evaluated by differential co-sedimentation at high (300 000 *g*) and low (17 000 *g*) centrifugation speeds, respectively.^27^ All five mutants retained F-actin binding ability (Fig. 1d, Table 1), while the L478P mutation in ABD2 completely abolished the ability of PLS3 to bundle actin filaments (Fig. 1e), likely due to the proximity of the mutated residue to the F-actin binding site of CH3 (Fig. 1b).^31^

**Fig. 2:**
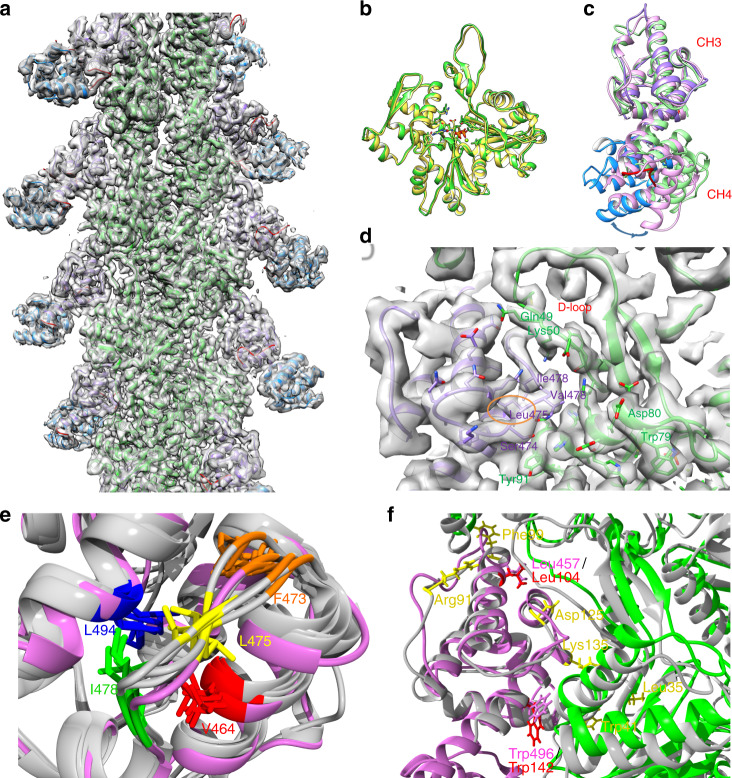
OI-linked L478P PLS3 mutation disrupts the actin-binding surface of ABD2. **a** Cryo-EM reconstruction with aligned model of ABD2_PLS2_-decorated F-actin (PDB ID: 6VEC; EMDB: EMD-21155). Actin is colored in green, CH3 and CH4 are in purple and blue, respectively. The loops linking the CH domains (385–394 and 511–517) are in red (see also Supplementary Fig. S2 and Supplementary Table S1). **b** Superposition of F-actin decorated by ABD2_PLS2_ (green; PDB ID: 6VEC) and pure F-actin (yellow; PDB ID: 5ONV). **c** Superposition of ABD2_PLS2_ in the complex with F-actin (CH3 domain is in purple, CH4 domain is in blue; PDB ID: 6VEC) and crystal structures of fimbrin ABD2 from *Arabidopsis thaliana* (pink; PDB ID: 1PXY) and *Schizosaccharomyces pombe* (green; PDB ID: 1RT8). The arrow indicates the rotation of CH4 upon F-actin binding. **d** The interface of the PLS2 CH3 domain (purple; PDB ID: 6VEC) binding to F-actin, showing that L475 (corresponding to L478 in PLS3) is not directly interacting with F-actin (green). **e** Superposition of the L475-containing loop of WT-ABD2_PLS2_ (in magenta) with the corresponding elements of ABDs from various t-CH-domain proteins (in gray): utrophin, dystrophin, filamin A, α-actinin, and ABD2 of yeast fimbrin (PDB IDs are provided in the Supplementary Table S2). Distances between L475 and the other four members of the cluster (V464, F473, I478, and L494) are consistent with hydrophobic interactions (see Supplementary Table S2). For respective multiple sequence alignment^87^ see Supplementary Fig. S3. **f** Superposition of F-actin-bound structure of ABD2_PLS2_ obtained in the present study (purple; PDB ID: 6VEC) with filamin A structure (gray; PDB ID: 6D8C) produced by aligning actin (gray/green for ABD2_PLS2_/filamin A). Actin-binding residues in the ABD of filamin A^33^ that are not conserved with PLS2 ABD2 are shown in yellow. The only two actin-binding residues of filamin conserved in PLS2 ABD2 are in red; the respective conserved PLS2 residues are in magenta

To gain structural insight into the effects of this bundling-deficient mutation, we obtained a 3.9-Å resolution cryo-EM map of F-actin decorated by the ABD2 domain of plastin 2 (PLS2) using a single-particle method for reconstruction of helical filaments^32^ (Fig. 2a, Supplementary Fig. S2, Supplementary Table S1). The use of PLS2 ABD2 for the reconstruction was justified by (i) an inherent instability of PLS3 ABD2 rendering its purification unfeasible^27^ and (ii) a high overall level of sequence conservation in ABD2 of the two isoforms and 100% identity in the 474–483 loop (PLS3 numeration) encompassing the L478P mutation (Supplementary Fig. S3). In the obtained cryo-EM reconstruction, the core of filamentous actin has the highest resolution, but the PLS2 CH3 and CH4 domains are both well resolved in the map. The resolution is lower the further from the helical axis, especially in the CH4 domain, and is shown with the local resolution estimation (Supplementary Fig. S2). The CH3 domain is directly interacting with F-actin, while CH4 domain has no contacts with F-actin. The superposition of F-actin in our reconstruction with a previously published pure F-actin structure (ADP state, PDB ID: 5ONV) shows that the RMSD of Cα atoms is only 0.8 Å (Fig. 2b), which indicates there are no significant conformational changes in F-actin after the decoration by PLS2. In comparison with the crystal structures of the yeast and plant fimbrin cores,^30^ the CH4 domain in the PLS2 ABD2/F-actin complex is shifted outward to prevent it from clashing with F-actin (Fig. 2c). The loop 474–483_PLS3_ (471–480_PLS2_; hereafter, PLS2 numeration is provided in parentheses) encompassing the L478P mutation makes extensive contacts with the D-loop and the 81–96 helix of actin located in subdomains 2 and 1, respectively (Fig. 2d). The side chain of L478 (L475) does not form direct contacts with actin as it points away, towards the ABD2 core. Essentially, the side chain of L478 (L475) is involved in stabilization of the loop structure by forming a hydrophobic cluster with conserved V467 (V464), F476 (F473), I481 (I478), and L497 (L494) residues (Fig. 2e, Supplementary Fig. S3, Supplementary Table S2). All these residues are conserved not only across ABD2 domains of different plastins/fimbrins, but also across ABDs of t-CH proteins from filamin, α-actinin, spectrin, dystrophin, and utrophin families (Supplementary Fig. S3), where they contribute to the conformational stability of the respective loop (Fig. 2e). The L478P mutation is likely to distort this cluster, thus influencing the loop conformation and negatively affecting binding of t-CH proteins to actin. On the other hand, of the nine residues of filamin A implicated in direct interaction with actin,^33^ only two are conserved in ABD2 of plastins (Fig. 2f, Supplementary Fig. S3), pointing to a considerable plasticity of the t-CH interaction mode with F-actin.

### Four OI-linked PLS3 mutations impair Ca^2+^ sensitivity

Ca^2+^ is recognized as a major signal transduction factor defining the sensitivity of osteocytes to mechanical stimuli in vitro and in vivo^34–38^ and is also involved in negative regulation of the actin-bundling ability of plastins. Ca^2+^ ions bind to two EF-hands in the RD of the protein, whose location relative to the actin-binding core remains unknown. It is recognized, however, that upon binding to Ca^2+^, the EF-hands wrap around the adjacent CBM,^27,39^ leading to inhibition of ABD2 via an unknown mechanism.

Under saturating Ca^2+^ concentrations, the A253_L254insN responded to Ca^2+^ similarly to WT PLS3, while actin bundling by N446S was inhibited by Ca^2+^ about two times more effectively (Supplementary Fig. S4, Table 1). The N446S mutation is associated with a strong OI phenotype in a heterozygous female patient^5^ and, therefore, is likely dominant. Remarkably, both mutants showed about an order of magnitude increased sensitivity to Ca^2+^ (pCa_50%_ 6.66 ± 0.17 and 6.44 ± 0.21) compared with WT PLS3 (pCa_50%_ 5.46 ± 0.14), as detected by a change in light scattering of actin-PLS3 bundles disassembled upon titration with Ca^2+^ (Fig. 1f, Table 1). The increased sensitivity of both mutants matches the K_d_ of Ca^2+^ to the high-affinity PLS3 EF-hand (K_d_ 0.37 µmol·L^−1^,^40^ corresponding to pCa 6.43), tentatively suggesting that the mutations diminish the contribution of the low-affinity EF-hand (K_d_ 10.6 µmol·L^−1^,^40^ corresponding to pCa 4.97) to plastin inhibition.

In contrast, the E249_A250insI-L and A368D mutants virtually lost sensitivity to Ca^2+^, enabling bundling even in the presence of high Ca^2+^ concentrations (Fig. 1f, Supplementary Fig. S4, Table 1). Also, regardless of the presence of Ca^2+^, the bundling ability of the E249_A250insI-L mutant was enhanced compared with that of WT PLS3 (Fig. 1e, Supplementary Fig. S4, Table 1). Given that all four mutations are located away from the EF-hands, perturbed interaction of RD with the ABD1–ABD2 core is a likely reason of the impaired Ca^2+^ sensitivity.

### OI-linked PLS3 mutants show differential subcellular distribution

**Fig. 3:**
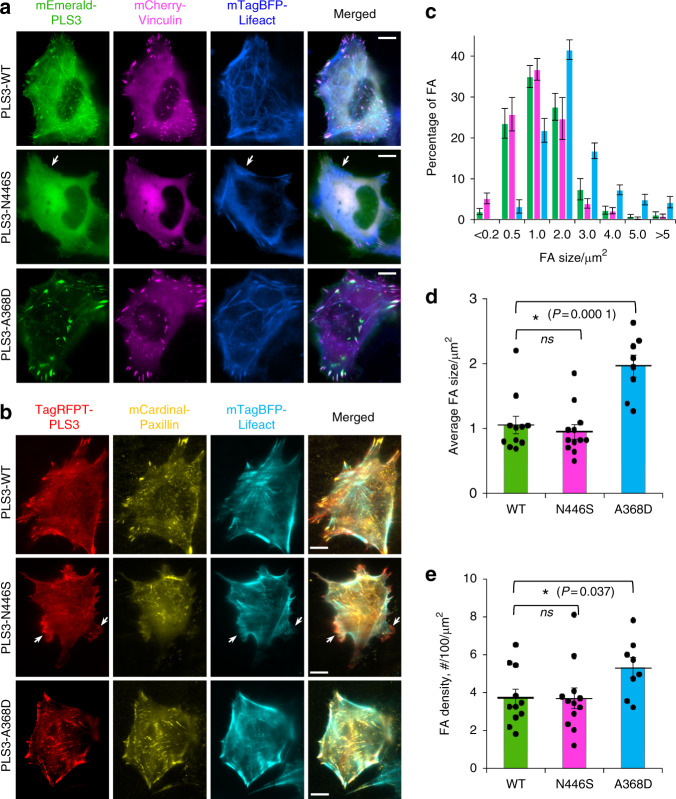
Effects of OI-linked PLS3 mutations on cellular localization of PLS3 and morphology of focal adhesions. **a** Live-cell total internal reflection fluorescence microscopy (TIRFM) imaging of U2OS osteoblasts co-transfected with mEmerald-PLS3 constructs (green), mCherry-vinculin (FA, magenta) and mTagBFP-Lifeact (F-actin, blue). See also Supplementary Fig. S5a. **b** Live-cell TIRFM imaging of Ocy454 osteocytes co-transfected with TagRFPT-PLS3 constructs (red), mCardinal-paxillin (FA, yellow), and mTagBFP-Lifeact (F-actin, cyan). See also Supplementary Fig. S5b. White arrows in (**a**, **b**) point at membrane ruffles; scale bars are 10 μm. FA size (area) distribution (**c**), average FA sizes (area; (**d**)), and FA density (number of FA per cell area; (**e**)) were quantified in U2OS cells transiently transfected with mEmerald-PLS3 constructs and immunostained with anti-vinculin (for FA) and phalloidin (for F-actin). The color legend in **c** is the same as in **d** and **e**. See also Supplementary Figs. S6 and S7. Number of analyzed cells is 11 for WT, 12 for N446S, and 8 for A368D (with 37–142 individual FAs per cell). Data are shown as means ± SE, individual data points are plotted as circles. Statistical significance was determined by two-sample (equal variances) Student’s *t* test with two-tailed distribution, *ns* is non-significant (*P* > 0.05)

Although PLS3 is ubiquitously expressed, all known developmental defects linked to PLS3 mutations are restricted to connective tissues: bones^5–17^ and fascia.^26^ Therefore, we focused on osteoblast (U2OS), osteocyte (Ocy454), and fibroblast (XTC) cell lines for PLS3 localization studies. In agreement with previous reports,^20,21,41,42^ mEmerald- or TagRFPT-tagged WT PLS3 was co-localized with the F-actin-rich elements at the cell edge, focal adhesions (FAs), and stress fibers (SFs) (Fig. 3a, b) of U2OS osteoblasts and Ocy454 osteocytes, while OI mutants displayed differential subcellular distribution, grouped in accordance with their biochemical properties. Specifically, the L478P bundling-deficient mutant showed diffuse localization, suggesting that binding via ABD1 alone is not sufficient to support its strong association with F-actin in any of the observed compartments (Supplementary Fig. S5). Both Ca^2+^-hypersensitive mutants showed reduced localization to FAs and SFs, but remained associated with the cell edge (Fig. 3a, b, Supplementary Fig. S5). Cells expressing these mutants displayed more prominent membrane ruffles. In striking contrast, both Ca^2+^-insensitive mutants vividly co-localized with FAs and SFs, whose morphology was both enhanced and distorted (Fig. 3a, b, Supplementary Fig. S5). Thus, the density and size of the FAs were significantly increased in cells expressing both Ca^2+^-insensitive mutants, while Ca^2+^-hypersensitive OI mutants of PLS3 did not affect these parameters (Fig. 3c–e, Supplementary Fig. S6). The expression levels of PLS3 constructs were comparable in all cases (Supplementary Fig. S7) and could not account for the observed differences.

**Fig. 4:**
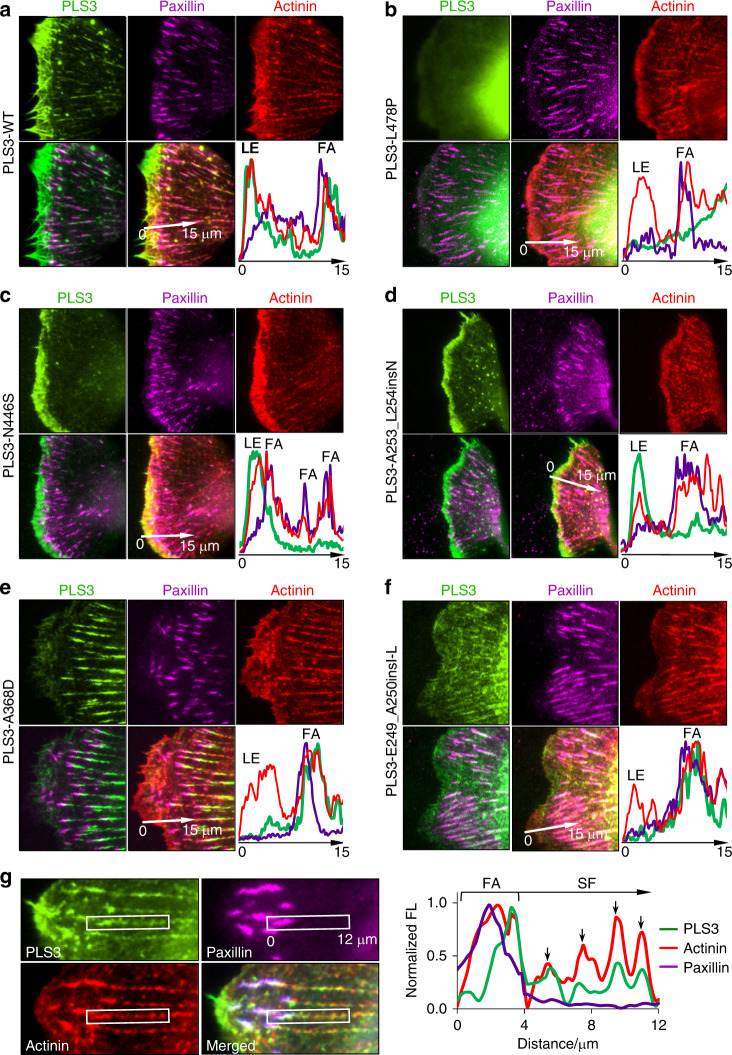
Differential distribution of OI-linked PLS3 mutants in spreading XTC fibroblasts. **a**–**f** Localization of PLS3 variants at the cell periphery was assessed by TIRFM in live XTC cells co-transfected with mEmerald-PLS3 constructs, mCardinal-paxillin, and mCherry-actinin. For each PLS3 construct, sets of individual channel images along with two-channel (PLS3 and paxillin) and three-channel (PLS3, paxillin, and actinin) overlays are shown. Graphs represent fluorescence intensity profiles along the 15-μm-long lines indicated by white arrows; plot colors match those of the corresponding fluorescent channels. Note differential distribution of OI PLS3 mutants between the leading edge (LE) and FAs. **g** Co-localization of mEmerald-PLS3-WT with FAs (mCardinal-paxillin) and SFs (mCherry-actinin) in cells with high levels of expression cultured in Ca^2+^-free medium. In the boxed areas, identical horizontal 12-μm-long lines were drawn (centrally, along the long axis) in each channel starting from the FA through the actin SF. Line intensity profiles show co-localization of PLS3 with actinin patches on the SFs and at the FAs (note that PLS3 localization is shifted towards the cell center)

The observed phenotypes of PLS3 mutants were reproduced in spreading *Xenopus laevis* XTC fibroblasts, whose flat morphology, prominent lamellipodia, and high resistance to photodamage^43^ make them particularly valuable for high-resolution live-cell microscopy. In XTC fibroblasts, mEmerald-tagged WT PLS3 was enriched at the leading edge, but also found moderately associated with FAs and SFs (Fig. 4a). Similarly to U2OS and Ocy454 cells, the bundling-deficient L478P mutant showed diffuse localization (Fig. 4b), whereas the Ca^2+^-hypersensitive mutants were highly enriched at the leading edge and almost excluded from the FAs and SFs (Fig. 4c, d). The Ca^2+^-insensitive mutants were depleted at the leading edge but strikingly enriched at FAs (Fig. 4e, f), which were notably larger than those in cells expressing WT or Ca^2+^-hypersensitive PLS3 mutants. Localization of PLS3 at the FA sites was partially overlapping with FA marker paxillin but extended toward the cell center; such a pattern was most reminiscent of the distribution of another t-CH-domain actin bundler α-actinin (Fig. 4g).

**Fig. 5:**
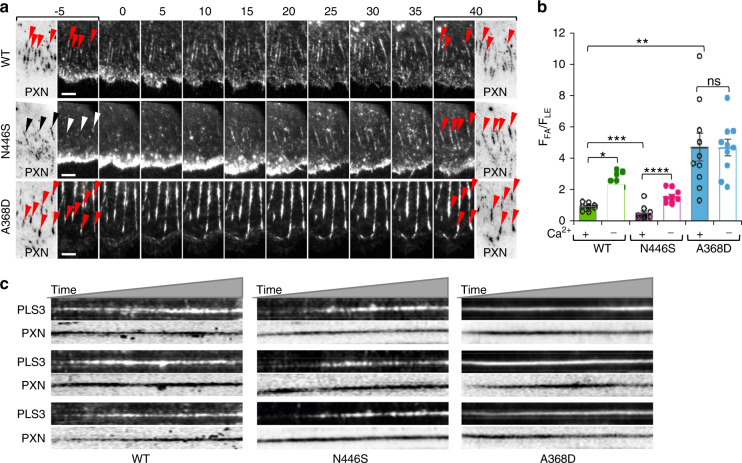
Effects of Ca^2+^ depletion on PLS3 redistribution to focal adhesion sites. **a** Time-lapse TIRFM images of XTC cells co-transfected with representative Ca^2+^-hypo- and hypersensitive mEmerald-PLS3 constructs and mCardinal-paxillin were recorded upon chelation of Ca^2+^ in the culture medium with 5 mmol·L^−1^ EGTA. Time before and after the addition of EGTA is indicated in minutes. mCardinal-paxillin is shown on inverted images (black signal on white background) marked PXN. Red arrowheads point to PLS3-positive FAs; black and white arrowheads denote PLS3-negative FAs. Scale bar is 5 μm (see also Supplementary Movie S1). **b** Ratios of PLS3 fluorescence at focal adhesions (F_FA_) to that of at the leading edge (F_LE_) in the Ca^2+^-containing medium (final extracellular concentration of Ca^2+^ 3.5 mmol·L^−1^) and upon Ca^2+^ chelation by 5 mmol·L^−1^ EGTA. Data are shown as means ± SE, individual data points are plotted as circles. Number of analyzed cells is *n* = 10 for each condition. Statistical significance was determined by two-sample (equal variances) Student’s *t* test with two-tailed distribution: *P = 1.4 × 10^−^^2^, **P = 4.5 × 10^−^^4^, ***p = 8.9 × 10^−^^5^, ****P = 2.6 × 10^−^^5^, *ns* is non-significant (*p* > 0.05). **c** Representative kymographs (length-time scans) display PLS3 and paxillin (PXN) fluorescence intensities over time along the lines drawn through individual FAs upon Ca^2+^ chelation with 5 mmol·L^–1^ EGTA

Considering the differential subcellular distribution of Ca^2+^-hyper- and hyposensitive PLS3 OI mutants, Ca^2+^ appeared to be a key factor regulating recycling of PLS3 from actin bundles at the FA sites to the branched network of actin filaments at the leading edge of spreading/migrating cells. To verify this hypothesis, we examined the dynamics of WT PLS3 and representative PLS3 OI mutants upon depletion of intracellular Ca^2+^ caused by the addition of EGTA to the medium. Notable redistribution of PLS3 from the leading edge to FAs was observed for both WT and N446S Ca^2+^-hypersensitive mutant, with the only difference that the leading-edge depletion was more prominent for WT PLS3, while FA enrichment was more notable for the N446S mutant (Fig. 5, Supplementary Movie S1). A moderate enrichment at the FA sites was visually detectable for the hyposensitive A368D mutant (Fig. 5a, b), but was found to be statistically insignificant (Fig. 5c).

### Mapping the position of the RD on the surface of PLS3 core

**Fig. 6:**
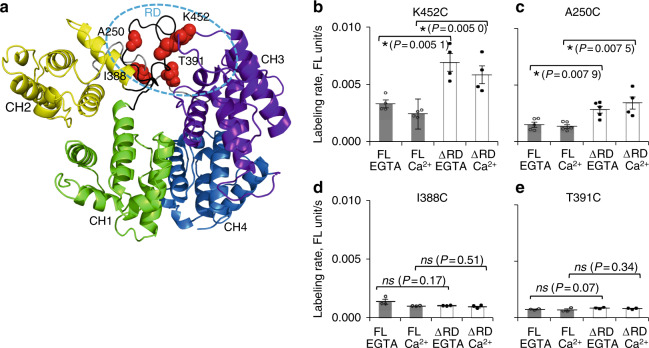
Mapping the position of the regulatory domain on the surface of PLS3 core. **a** Positions of single-cysteine mutations in the loops harboring OI-associated mutations (A250C in the 240–268 and K452C in the 446–456 loops) and in the ABD1–ABD2-connecting linker (I388C and T391C in the 377–394 linker) are shown in red on a homology-based model of PLS3 (Phyre2^86^; color scheme as in Fig. 1). The proposed position of RD is encircled by a dashed line. **b**–**e** Acrylodan labeling of the cysteine residues described in (**a**) introduced as the only cysteines into the full-length PLS3 (FL) and PLS3 lacking the Ca^2+^-binding regulatory domain (ΔRD). Labeling was assessed for each construct in the presence of either 1 mmol·L^−1^ EGTA or CaCl_2_. The rates were calculated by fitting the data to a pseudo-first order kinetic model and plotted as means ± SE (*n* = 4 for **b**, *n* = 5 for **c**, *n* = 3 for **d**, **e**); individual data points are plotted as circles. Statistical significance was determined by two-sample (equal variances) Student’s *t* test with two-tailed distribution

To date, high-resolution structures of full-length plastin/fimbrin do not exist, and, therefore, the location of RD relative to the core is unknown and the mechanism of plastin inhibition by Ca^2+^ is not understood. Remarkably, all four PLS3 OI mutations with impaired Ca^2+^ sensitivities are grouped near the same face of the ABD core (Fig. 1b), while two of them are even located in the same CH1–CH2 loop (E249_A250insI-L and A253_L254insN). We speculated that the perturbed Ca^2+^ sensitivity of the four mutants suggests that the mutations are at or near the RD/core interface (Figs. 1b and 6a, dashed oval). To test this hypothesis, we used full-length and RD-truncated (ΔRD) PLS3 constructs to evaluate solvent accessibility of single-cysteine residues incorporated into the core loops facing the tentative RD-binding region (Fig. 6a, dashed oval) and involved in osteoporosis (i.e., 240–268 and 446–456 loops harboring E249_A250insI-L, A253_L254insN, and N446S OI mutations). Single-cysteine PLS3 mutants K452C and A250C in constructs containing RD incorporated a Cys-selective acrylodan probe approximately two times slower than their counterparts without RD (Fig. 6b, c), consistent with a reduced solvent accessibility of the loops due to their proximity to RD. Ca^2+^ did not affect the modification rates of A250C and only mildly influenced the modification of K452C, suggesting that the Ca^2+^-induced conformational rearrangements only moderately affect the RD position.

In addition to the osteoporosis loop A250C and K452C mutants, we also tested the I388C and T391C constructs with lone cysteines located in a long linker loop between ABD1 and ABD2 (i.e., 377–394 loop; Fig. 6a). Labeling of both I388C and T391C with acrylodan was very slow regardless of the presence of RD (Fig. 6d, e) suggesting their overall buried position.

## Discussion

### F-actin bundling is the primary physiological function of plastins

The majority of the OI-linked PLS3 mutations are either nonsense or frameshift mutations resulting in premature termination codons. Such mutations rarely result in translated protein products due to nonsense-mediated mRNA decay, a quality control measure that degrades mRNA containing premature stop-codons preventing the generation of truncated proteins.^28,29^ While mRNAs can avoid nonsense-mediated decay,^44^ none of the canonical mechanisms apply to the majority of the identified OI mutations. Accordingly, the two studies that evaluated the in vivo expression of truncated OI PLS3 mutants, did not detect protein products.^13,16^ Furthermore, were the nonsense and frameshift mutants to be translated, they would not be fully functional as only three would retain protein sequence through CH3 domain, while CH4 domain also contributes to F-actin bundling.^27^ Our analysis confirmed substantial destabilization and aggregation/denaturation for the longest truncated OI PLS3 variants, which could be produced due to the frameshift mutations T578NfsX4 and A589QfsX21 introducing premature termination codons 49 and 20 amino acids before the PLS3 natural stop codon.

Therefore, in this study we focused on the five missense and insertion OI PLS3 mutations that are expected to produce full-length protein products and, according to our DSF data, do not result in substantial protein destabilization. Of these five mutants, L478P lacks the bundling ability and, therefore, is functionally most similar to the OI-linked PLS3 deletion and truncation mutants and most clearly connected to the disease phenotype. Plastins can bind to F-actin via their ABD1 domain independent of bundling^27^ and ABD1 alone is sufficient to positively affect Arp2/3-mediated actin dynamics in vitro.^45^ Possible explanations for this observation are a competitive replacement of tropomyosin enabling a higher filament recycling rate by cofilin,^46^ or, on the contrary, stabilization of filaments via competition with cofilin.^45^ Yet, despite the unperturbed binding of L478P PLS3 via ABD1 to actin, the mutation still leads to osteoporosis and appears to be insufficient for the association of the mutant with either FAs or the lamellipodia (Figs. 3 and 4). Plastins are well recognized to stabilize parallel aligned F-actin bundles.^47^ Of the two major PLS3-enriched cellular structures, FAs contain parallel actin bundles, while the leading-edge actin cytoskeleton is enriched in branched actin networks depleted in parallel bundles. It remained unclear, until now, whether plastins are associated with the leading-edge actin cytoskeleton in an F-actin binding mode (i.e., through ABD1) or in a bundling mode (i.e., through both ABD domains). These data correlate with the essential role of fimbrin’s (a yeast plastin orthologue) actin-bundling activity in endocytosis at the actin patches,^46,48^ which originate as the Arp2/3 complex-organized branched actin networks. Our findings suggest that binding via both ABD1 and ABD2 is essential for PLS3 localization both at the leading edge and FAs and corroborate that F-actin bundling is the primary physiological function of plastins at both locations.

### OI mutations unbalance PLS3 regulation by Ca^2+^

Despite the well-recognized role of Ca^2+^ in plastin regulation in vitro, how the EF-hands domain is positioned on the core is unclear, and the mechanism of the inhibition is not understood. We found that two of the five characterized OI-causative PLS3 mutants lost Ca^2+^ sensitivity and remained constitutively active even in the presence of Ca^2+^, while two others became more sensitive to Ca^2+^. While the former is a typical ‘loss of function’ effect, as the RD of plastin loses its ability to inhibit bundling in the presence of Ca^2+^, the latter ‘gain of function’ effect (a higher sensitivity to Ca^2+^) is much less common and calls for at least a speculative explanation. In contrast to PLS2, whose both EF-hand domains share the same ~1-µmol·L^−1^ K_d_ to Ca^2+^, PLS3 EF-hands vary in their affinity to Ca^2+^ by nearly thirty-fold.^40^ The measured sensitivity levels of the hypersensitive mutants (pCa_50%_ = 6.5–6.7) match the 0.37-µmol·L^−1^ K_d_ of the high-affinity binding PLS3 EF-hand (~pCa 6.4). This observation tentatively suggests that while Ca^2+^ binding to both EF-hands is required for inhibition of WT PLS3, single high-affinity site binding is sufficient to inhibit the hypersensitive mutants. It is tempting to speculate that a similar effect, achieved via a posttranslational modification or caused by a binding partner, may represent a physiological mechanism of tuning PLS3 Ca^2+^ sensitivity. Interestingly, the abnormally high level of PLS3 Ca^2+^ sensitivity leading to osteoporosis is nearly identical to the physiological sensitivities of PLS2 and PLS1,^27^ indicating that the Ca^2+^ regulation of the isoforms is tuned to their tissue-specific functions.

### OI mutations point on the location of the RD

The 240–268 and 446–456 loops harboring three of the mutants with perturbed Ca^2+^ sensitivity, although belonging to different ABDs, are located on the same surface within ~20 Å of each other (Fig. 1b), a distance that can be easily bridged by RD (~35 Å). The proposed arrangement is consistent with covalent crosslinking of RD to both ABD1 and ABD2.^27^ Similarly, the presence of RD limits solution accessibility of the loops as deduced from less effective acrylodan labeling of cysteine residues embedded into the loops (Fig. 6b, c), further supporting proximity of the osteoporosis loops to the RD. The fourth mutation (A368D), although not in these loops, faces both the 224–239 helix (preceding the 240–268 loop) and the 123–137 helix (following the linker between RD and ABD1) and, therefore, may unbalance Ca^2+^ regulation by affecting both RD and an RD-binding site on the 240–268 loop. The unstructured nature of the 240–268 loop^30^ also indirectly supports its involvement in binding to the RD. Indeed, disordered protein regions are notoriously engaged in polymorphic interactions^49^ and may effectively mediate binding to several partners^50^ or to different conformational states of the same partner such as the Ca^2+^-free and -bound states of RD.

### Mechanistic model of the Ca^2+^-dependent regulation of PLS3 activity

**Fig. 7:**
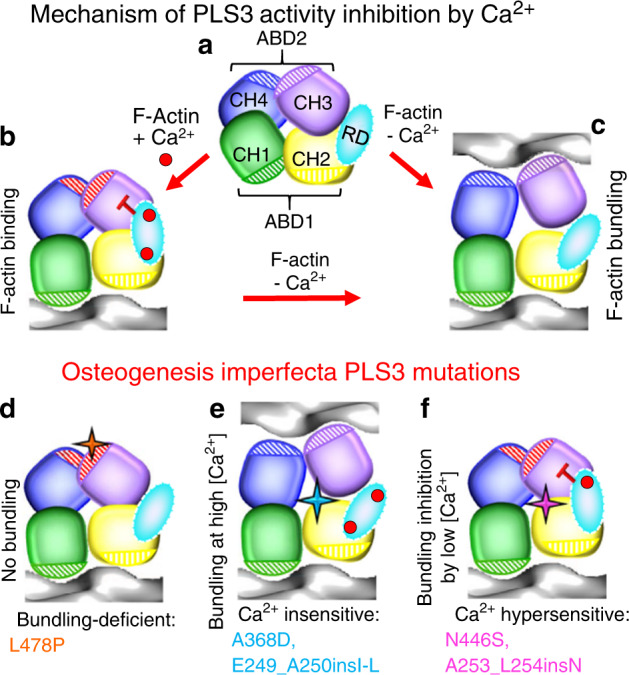
Model of plastin regulation by Ca^2+^. **a** Schematic representation of plastin domains (the color scheme and domain designation as in Fig. 1). **b** In the presence of Ca^2+^, plastins bind to actin via ABD1 only, while CH3–CH4 of ABD2 are locked in the inhibited state (red dashed) by stapling ABD2 to ABD1 by RD. **c** Dissociation of Ca^2+^ releases the RD-applied constraints to ABD2 allowing its binding to actin and resulting in F-actin bundling. The OI PLS3 mutations lead to osteoporosis by either directly disrupting the actin-binding site of ABD2 (L478P; (**d**)), inhibiting the ABD2 by sub-physiological Ca^2+^ concentrations (N446S and A253_L254insN; (**f**)), or by disabling the inhibition by physiological Ca^2+^ concentrations (A368D and E249_A250insI-L; (**e**)), preventing normal functions of PLS3

Proximity of the linkers connecting the CH domains to the proposed RD-binding site (Fig. 6a) suggests that RD may affect F-actin binding by restraining the relative orientation of the CH domains within ABD2 (CH3 to CH4), between ABDs (CH2 to CH3), or both. Based on the results of the current and recent studies, we propose the following model of plastin regulation by Ca^2+^ (Fig. 7). In the presence of Ca^2+^, plastins bind to actin via ABD1 only,^27^ while CH3–CH4 are locked in the inhibited state by RD, which restricts the rearrangements required for the CH3 binding to actin (Fig. 2c) by fastening ABD2 to ABD1. Dissociation of Ca^2+^ leads to separation of the EF-hands from the CBM, unwinding of the CBM helix to a relaxed structure,^39^ and release of the ABD1–ABD2 constraint. In line with this model, the OI PLS3 mutations lead to osteoporosis by either (i) directly disrupting the actin-binding site of ABD2 (L478P), (ii) inhibiting the interaction by low, non-inhibitory for WT PLS3, Ca^2+^ concentrations (N446S, A253_L254insN), or (iii) by disabling the inhibition by physiological Ca^2+^ concentrations (A368D, E249_A250insI-L). Such dysregulation leads to either weakening or, on the contrary, nonphysiological enforcement of FA sites by respectively accelerating or preventing the PLS3 recycling to the cell periphery.

### Links between PLS3 function, calcium signaling, and osteoporosis

Although the exact role of PLS3 in osteogenesis is currently unclear, numerous indirect lines of evidence suggest its contribution to sensing and transduction of mechanical stimuli. Of the three cell types immediately involved in constructing and remodeling the bone, the role of PLS3 in bone resorption by osteoclasts is rather convoluted, given that the major plastin isoform in these cells is PLS2 and the presence of PLS3 was not clearly demonstrated. Nevertheless, a recent work has proposed a link between PLS3, NFκB signaling, and the regulation of osteoclast activity.^51^ Another study has found no effect of PLS3 mutations on the osteoclast activity.^14^

In contrast, PLS3 is the predominant isoform in the other two major bone cell types, osteoblasts and osteocytes. Moreover, its expression in osteoblasts is increased upon maturation and PLS3 is highly enriched at the bifurcations of osteocyte dendritic processes,^52^ which are recognized as mechanotransducing elements.^52,53^ The ability of osteoblasts and especially osteocytes to sense and respond to mechanical stimuli is the key factor defining bone morphogenesis and remodeling directly (by affecting the synthetic activity of osteoblasts) and indirectly (by controlling the activity and apoptosis of both osteoblasts and osteoclasts by humoral factors).^54–56^

In osteoblasts and osteocytes, rise of cytoplasmic Ca^2+^ is linked to bone development via calcineurin/NFAT^57^ and noncanonical Wnt^53^ pathways, the latter of which is recognized as a central signaling pathway in osteogenesis. Furthermore, the rise of cytoplasmic Ca^2+^ in these cells can be caused by mechanical stimuli,^37,58^ which is an essential component of mechanically controlled bone formation and remodeling by both osteoblasts and osteocytes. Although the mechanisms of osteoblast/osteocyte mechanosensitivity are debated, integrin/adhesion-dependent activation of voltage-gated calcium channels are thought to be involved.^37,58–60^ Association of plastins with integrin-based FAs and its sensitivity to Ca^2+^ furthers the link between PLS3 and the mechanotransduction machinery. Local Ca^2+^ spikes associated with FAs are recognized in other cell types (e.g., fibroblasts^61^ and astrocytes^62^) and may represent a common mechanism of cell response to mechanical stimuli. In astrocytes, this results in accelerated recycling of FA sites via recruitment of focal adhesion kinase (FAK).^63^ The importance of FAK and FA dynamics in bone development is illustrated by a potent suppression of mechanically induced bone regeneration in FAK-deficient mice.^64^ Our finding that Ca^2+^ promotes dissociation of PLS3 from FAs and SFs, allowing its recycling to the leading edge, may represent a parallel, FAK-independent mechanism of FA dynamics regulation.

Overall, the reduced or increased sensitivity to Ca^2+^ of four out of five OI-associated PLS3 mutations characterized in the present study as well as their respectively perturbed localization in osteoblast, osteocyte, and fibroblast cells indicate that the delicately controlled Ca^2+^-dependent regulation of actin bundling by PLS3 is essential for normal bone formation.

## Materials and methods

### Plasmid construction

Recombinant plastins were cloned as N-terminally 6xHis-tagged constructs into a modified pColdI vector (Clontech) containing a TEV protease recognition site^27^ using NEBuilder HiFi DNA Assembly Master Mix (New England Biolabs). Site-directed mutagenesis was carried out based on Quick-change Site-directed Mutagenesis strategy (Agilent Technologies) using Q5 DNA polymerase and DpnI restriction enzyme (New England Biolabs). QuikChange Lightning Multi Site-Directed Mutagenesis Kit (Agilent Technologies) was used for multisite mutagenesis.

### Protein expression and purification

Actin was purified from chicken skeletal muscle acetone powder obtained from flash-frozen chicken breasts (Trader Joe’s)^65^ as previously described.^66^ Actin was stored up to 1 month on ice in G-buffer (5 mmol·L^−1^ Tris-HCl [pH 8.0], 0.2 mmol·L^−1^ CaCl_2_, 0.2 mmol·L^−1^ ATP, 5 mmol·L^−1^ β-mercaptoethanol, 0.1 mmol·L^−1^ phenylmethanesulfonyl fluoride [PMSF], and 0.01% NaN_3_) with dialysis against fresh G-buffer after 2 weeks.

Plastins were expressed in BL21-CodonPlus(DE3) *E. coli* (Agilent Technologies) grown in nutrient-rich media (1.25% tryptone, 2.5% yeast extract, 125 mmol·L^−1^ NaCl, 0.4% glycerol, and 50 mmol·L^−1^ Tris-HCl [pH 8.2]). Upon reaching the OD_600_ of 1–1.2, the cells were cooled to 15 °C and expression was induced by the addition of 1 mmol·L^−1^ isopropyl β-D-1-thiogalactopyranoside. Cells were maintained at 15 °C for 16 h–24 h before harvest by centrifugation. Plastin mutants were purified by immobilized metal affinity chromatography on TALON cobalt resin (Clontech) under native conditions. Elution was carried out by the addition of 250 mmol·L^−1^ imidazole, 50 mmol·L^−1^ 4-(2-hydroxyethyl)-1-piperazineethanesulfonic acid (HEPES; pH 7.4), 300 mmol·L^−1^ NaCl, and 0.1 mmol·L^−1^ PMSF. Purified plastins were dialyzed overnight against PLS buffer (10 mmol·L^−1^ HEPES [pH 7.0], 30 mmol·L^−1^ KCl, 2 mmol·L^−1^ MgCl_2_, 0.5 mmol·L^−1^ ethylene glycol-bis(β-aminoethyl ether)-N,N,N′,N′-tetraacetic acid [EGTA], 2 mmol·L^−1^ dithiothreitol [DTT], and 0.1 mmol·L^−1^ PMSF) before being flash-frozen in liquid nitrogen and stored at −80 °C.

### Protein labeling with acrylodan

Single-cysteine mutations were introduced on the Cys-null background in the full-length and ΔRD PLS3 constructs. Proteins were reduced in the presence of 10 mmol·L^−1^ DTT for 1 h on ice followed by DTT removal by passing through a NAP5 desalting column (GE Healthcare) equilibrated with buffer containing 10 mmol·L^−1^ HEPES (pH 7.0) and 30 μmol·L^−1^ KCl. Labeling with acrylodan was carried out as previously described.^67^ Briefly, reduced PLS3 constructs were labeled under pseudo-first-order conditions with a 1:100 acrylodan to PLS3 molar ratio (0.05 mmol·L^−1^ acrylodan and 5 μmol·L^−1^ PLS3) in the buffer above with the addition of 1 mmol·L^−1^ either EGTA or CaCl_2_. Labeling rate was monitored at 25 °C using a PTI QuantaMaster 400 spectrofluorometer (Horiba) with excitation and emission wavelengths of 385 and 465 nm, respectively.

### Differential scanning fluorimetry (DSF)

DSF was conducted as reported previously.^68,69^ Briefly, plastins were diluted to 3 μmol·L^−1^ in PLS buffer in the presence of 1:5 000 dilution of Sypro Orange dye (Invitrogen). Changes in fluorescence of the dye, which preferentially binds to protein hydrophobic regions exposed upon temperature-induced unfolding, were measured in triplicates using a CFX Real-Time PCR Detection System (Bio-Rad). The melting temperatures (T_m_) were determined as the maximum of the first derivative of each normalized experimental curve and expressed as the mean of three independent repetitions ± standard error (SE).

### F-actin binding and bundling co-sedimentation assays

G-actin was switched from a Ca^2+^- to Mg^2+^-bound form by the addition of 0.1 mmol·L^−1^ MgCl_2_ and 0.5 mmol·L^−1^ EGTA and incubated on ice for 10 min. Subsequently, actin polymerization was induced by the addition of MgCl_2_, KCl, and HEPES (pH 7.0) to 2, 30, and 10 mmol·L^−1^, respectively. Polymerization was carried out for 30 min at room temperature before addition of plastin. In actin-binding assays, plastins were added to a final concentration of 5 µmol·L^−1^, and actin was varied from 1 to 50 µmol·L^−1^. For actin-bundling assays, actin was used at 5 µmol·L^−1^ while the plastin concentrations were varied from 0.1 to 15 µmol·L^−1^. Where indicated, CaCl_2_ was added to yield a concentration of free Ca^2+^ of 0.5 mmol·L^−1^ determined as described.^70^ Reactions were incubated overnight at 4 °C followed by 1 h at 25 °C prior to centrifugation. Binding reactions were centrifuged at 300 000 *g* and 25 °C for 30 min. Bundling reactions were centrifuged at 17 000 *g* and 25 °C for 15 min. Supernatants were separated from pellets and analyzed by SDS-PAGE stained with Coomassie Brilliant Blue. Densitometry analysis was performed using ImageJ software.^71,72^

K_d_s were determined using the binding isotherm equation:

$${\mathrm{Fraction}}\;{\mathrm{PLS}}\;{\mathrm{Bound}} = \frac{{P + A + K_d - \sqrt {\left( {P + A + K_d} \right)^2 \,-\, 4PA} }}{{2P}},$$

where *P* is the concentration of plastin and *A* is the concentration of F-actin.

Bundling efficiency was calculated by fitting to the Hill equation:

$${\mathrm{Percentage }}\;{\mathrm{of }}\;{\mathrm{Actin}}\;{\mathrm{Bundled}} = \frac{{[PLS]^n}}{{K_A^n + [PLS]^n}},$$

where *n* is the Hill coefficient and *K*_*A*_^*n*^ is the concentration of plastin at 50% actin bundled.

Binding and bundling experiments were conducted in at least three and two independent repetitions, respectively, and the final values were presented as mean values with SE or standard deviations (SD) as indicated in the figure and table legends.

### Light scattering assay

To determine Ca^2+^ sensitivity of PLS3 constructs, light scattering by PLS3-induced F-actin bundles was measured at 90° to the incident light using a PTI QuantaMaster 400 spectrofluorometer (Horiba). Both excitation and emission wavelengths were set at 350 nm with 3 nm slits. Actin and plastin (5 and 1 μmol·L^−1^, respectively) were mixed in PLS buffer and incubated overnight at 4 °C followed by incubation for 1 h at 25 °C and degassing for 15 min under vacuum. Ca^2+^ was increased incrementally and measurements were taken for at least 1 min between the titration points and averaged for each increment. The resulting values were normalized and the average of each of three independent experiments ± SE was plotted versus free pCa in solution. The pCa_50%_ values were calculated by fitting the data to a logistic curve using Origin software (OriginLab).

### Cryo-EM data collection, helical reconstruction, and model building

To prepare the F-actin/ABD2_PLS2_ complex, skeletal muscle actin was polymerized as previously described^31^; ABD2 of PLS2 was added at a fourfold excess to 1-μmol·L^−1^ F-actin. A 1.5 μL sample of the F-actin/ABD2 complex was applied to discharged lacey carbon grids, followed by plunge freezing with a Leica EM GP. Movies were collected in a Titan Krios cryo-electron microscope at 300 keV equipped with a Falcon III direct electron detector, sampling at 1.4 Å/pixel. The defocus range was set to 1.5–2.5 μm, with a total dose of ∼55 electrons/Å^2^. MotionCor2 was used to motion correct and dose-weight all the movies, followed by CTF estimation of the aligned images using the CTFFIND3 program.^73^ Images with poor CTF estimation were eliminated. The e2helixboxer program in the EMAN2^74^ software package was used for boxing the long filaments. Subsequently, the filaments were extracted and cut into shorter segments in Relion 3.0,^75^ which generated 248 184 256px-long overlapping segments. A subgroup (~49% of the full dataset) of nicely ABD2-decorated F-actin was selected after 2D and 3D classification. We further used AutoRefine followed by polishing, as well as CtfRefine in Relion 3.0. The final symmetry converged to twist of −166.5° and rise of 28.0 Å with local symmetry searches. The final resolution was determined by both map:map FSC as well as model:map FSC. The map:map FSC yielded a resolution of 3.9 Å at FSC = 0.143, and the model:map FSC also showed a resolution of 3.9 Å at FSC = 0.38 (which is √0.143).

We used a cryo-EM structure of actin in complex with ADP (PDB ID: 5ONV) and a PLS2 homology model based on a template (PDB ID: 1RT8) from SWISS-MODEL^76^ as initial models. The subunit models of actin and ABD2 of PLS2 were first fit as rigid bodies into the 3.9 Å cryo-EM map using UCSF Chimera,^77^ followed by model refinement using RosettaCM.^78^ The top ten models were selected and refined by COOT^79^ and PHENIX.^80^ The best model was then symmetrically built by using Rosetta, followed by PHENIX real space refinement. The final model was validated by MolProbity^81^ (Table S1). The cryo-EM map was deposited with accession code EMD-21155 in the Electron Microscopy Data Bank (EMDB), and the coordinates were deposited in the Protein Data Bank (PDB) with accession code PDB ID: 6VEC. Molecular graphics were performed using PyMol Molecular Graphics System (version 2) and UCSF Chimera.^77^

### Cell culture

U2OS human osteosarcoma cells (RRID:CVCL_0042) were maintained in DMEM supplemented with 10% fetal bovine serum (FBS; Corning), 100 μg·mL^−1^ streptomycin, 100 μg·mL^−1^ penicillin, 2 mmol·L^−1^ glutamine at 37 °C in a humidified atmosphere containing 5% CO_2_. Ocy454 osteocytic cells (RRID:CVCL_UW31)^82^ derived from a double transgenic mouse expressing GFP under the control of dentin matrix protein 1 promoter and SV40 large T antigen, were obtained from Bone Cells Core services (Boston University) and grown on plates coated with rat-tail collagen I (5 µg·cm^−2^; Corning) in α-MEM medium (Gibco) supplemented with 10% FBS, 100 μg·mL^−1^ streptomycin, 100 μg·mL^−1^ penicillin at 33 °C in a humidified atmosphere containing 5% CO_2_. *X*. *laevis* XTC-2 fibroblasts (RRID:CVCL_5610) were cultured in 70% L-15 medium containing 10% FBS, 100 μg·mL^−1^ streptomycin, 100 μg·mL^−1^ penicillin at 25 °C at ambient humidity and CO_2_. Human U2OS cell line was authenticated by microsatellite genotyping (Genomics Shared Resource, OSU Comprehensive Cancer Center) with 100% match using The Cellosaurus cell line database.^83^ All cell lines were mycoplasma-free as tested by PCR-based mycoplasma detection analysis.^84^

### Transient transfection

Plastin constructs fused at their C-terminus with either mEmerald or TagRFPT were cloned into a pcDNA3.1 vector (Invitrogen) using NEBuilder HiFi DNA Assembly Master Mix (New England Biolabs). mCherry-Alpha-Actinin-19, mCherry-Vinculin-23, mTagBFP-Lifeact-7, and mCardinal-Paxillin-22^85^ were gifts from Michael Davidson (Addgene 54975, 55159, 54596, and 56171, respectively). Transient transfections were carried out using Turbofect transfection reagent (Thermo Fisher Scientific) according to manufacturer instructions.

### Immunofluorescence

U2OS cells transiently transfected with mEmerald-PLS3 constructs were plated on 96-well ibiTreat μ-plate (ibidi 89626). After 24 h of transfection cells were fixed with 4% paraformaldehyde in phosphate buffered saline (PBS; Sigma) for 10 min, permeabilized with 0.1% of Triton X-100 in PBS for 5 min, blocked in 1% bovine serum albumin in PBS for 30 min, stained with rabbit anti-vinculin (Bethyl A302–535A-T; 1:200) followed by TRITC-conjugated anti-rabbit (Sigma T6778, 1:500), contra-stained with coumarin-phalloidin (Santa Cruz Biotechnology sc-301532; 0.2 μmol·L^−1^ final) and imaged using wide-field epifluorescence and Nikon CFI Plan Apochromat λ ×60 oil objective (NA 1.40) on a Nikon Eclipse Ti-E microscope. Vinculin-positive FAs were counted in PLS3-mEmerald-transfected cells (number of analyzed cells was 11 for WT PLS3, 12 for N446S PLS3, and 8 for A368D PLS3, with 37–142 individual FAs per cell) and sizes of individual FA sites were measured using ImageJ.^72^

### Western blotting

U2OS cells transfected with mEmerald-PLS3 constructs were harvested and cell lysates were prepared in the reducing SDS-PAGE sample buffer. Samples were subjected to PAGE and transferred to nitrocellulose. Membranes were blocked in PBS containing 0.1% Tween-20 and 5% nonfat dry milk for 1 h at room temperature and incubated with anti-PLS3 antibody (Sigma SAB2700266; 1:1 000) in the blocking buffer overnight at 4 °C. Following three washes with PBS containing 0.1% Tween-20, membranes were incubated with anti-rabbit antibody conjugated with horseradish peroxidase (HRP; Sigma A0545; 1:10 000) for 1 h at room temperature. Signal was detected using chemiluminescent HRP substrate WesternBright Sirius (Advansta) in an Omega Lum G imager (Aplegen).

### Live-cell imaging

XTC cells in six-well plates at 70% confluence were co-transfected with the mEmerald-PLS3 constructs, mCherry-actinin, and mCardinal-paxillin. Prior to live-cell imaging, transfected cells were trypsinized and plated on poly-D-lysine (PDL) coated coverslips (Neuvitro GG-25–1.5-PDL) in Attofluor chambers (Invitrogen) in 70% L-15 media lacking phenol red and FBS. Cells were allowed to spread for 30 min at 25 °C and imaged at ambient conditions using Nikon TIRF module and a CFI Plan Apochromat λ ×100 oil objective (NA 1.45) on a Nikon Eclipse Ti-E microscope equipped with perfect focus system. Laser power was set to 15% and the field diaphragm was partially closed to minimize the cell area exposure and photodamage during the image acquisition. For Ca^2+^ removal experiments, 5 mmol·L^−1^ EGTA was added to the culture medium to chelate Ca^2+^ and images were recorded with 30-s intervals. Image processing and assessment of the ratio of PLS3-mEmerald fluorescence signal at the cell edge and in FAs were performed using ImageJ.^72^ Kymographs and Supplementary Movie S1 were generated using Multiple Kymographs and Multi Stack Montage plugins of ImageJ, respectively.

U2OS and Ocy454 cells were plated on collagen I-coated PDL coverslips in phenol red-free serum-containing L-15 or α-MEM medium, respectively. U2OS cells were co-transfected with the mEmerald-PLS3 constructs, mCherry-vinculin, and mTagBFP-Lifeact, while GFP-positive Ocy454 cells were co-transfected with the TagRFPT-PLS3 constructs, mCardinal-paxillin, and mTagBFP-Lifeact. After 24 h of transfection, live-cell TIRFM imaging was conducted as described above.

### Statistical analysis

Each experiment was conducted in 2–5 independent repetitions and final values were presented as mean values with SE or SD as indicated in the corresponding figure/table legends alone with the exact number of the conducted experiments/quantified events (*n*). Two-sample (equal variance) Student’s *t* test with two-tailed distribution was used for data comparison (*P* values < 0.05 were considered statistically significant). Data were graphed using KaleidaGraph (Synergy Software) and Excel (Microsoft Office); Origin software (OriginLab) was used for data fitting.

## Supplementary information

Supplemental materials

Movie S1
