# Supplementary material for: Osteogenesis imperfecta mutations in plastin 3 lead to impaired calcium regulation of actin bundling
Source: Bone Res. 2020 May 22;8:21. doi: 10.1038/s41413-020-0095-2 (PMC7244493; doi:10.1038/s41413-020-0095-2)
Supplement: Supplementary file 1 — Supplemental materials [file 41413_2020_95_MOESM1_ESM.pdf]

Supplementary Information for

Osteogenesis imperfecta mutations in plastin 3 lead to impaired calcium regulation of actin bundling

Christopher L. Schwebach, Elena Kudryashova, Weili Zheng, Matthew Orchard, Harper Smith, Lucas A. Runyan, Edward H. Egelman, and Dmitri S. Kudryashov

Supplementary Figure S1

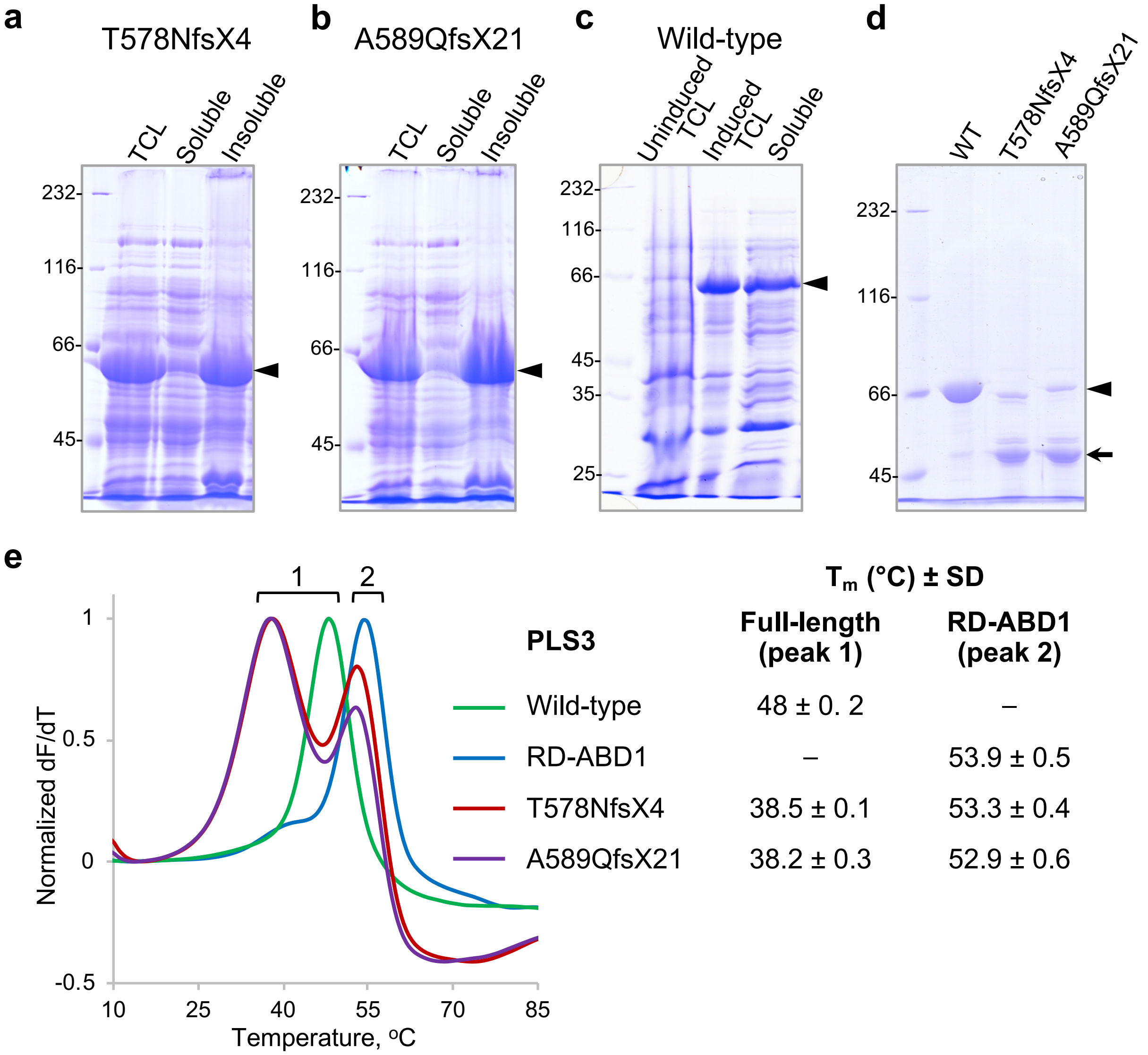

PLS3	T_m (°C) ± SD Full-length (peak 1)	RD-ABD1 (peak 2)
Wild-type	48 ± 0. 2	–
RD-ABD1	–	53.9 ± 0.5
T578NfsX4	38.5 ± 0.1	53.3 ± 0.4
A589QfsX21	38.2 ± 0.3	52.9 ± 0.6

Supplementary Figure S1. Frameshift OI PLS3 mutations T578NfsX4 and A589QfsX21 resulting in truncations of C-terminal 49 and 20 amino acids, respectively (see also **Fig. 1a**), lead to PLS3 protein destabilization. **a-c** SDS-PAGE of *E. coli* lysates reveals that T578NfsX4 (**a**; arrowhead) and A589QfsX21 (**b**; arrowhead) PLS3 variants are mainly insoluble upon expression, in contrast to the wild-type PLS3 (**c**; arrowhead). *E. coli* cells expressing PLS3 constructs were lysed in lysis buffer under native conditions (total cell lysate - 'TCL') and fractionated by centrifugation (supernatant - 'soluble' and pellet - 'insoluble' fractions). **d** Recombinant T578NfsX4 and A589QfsX21 PLS3 variants are unstable and become cleaved upon purification from the soluble fraction: a relatively minor full-length construct band (arrowhead) copurifies with a major band of a smaller molecular weight corresponding to a truncated N-terminal 6xHis-tag-containing RD-ABD1 product (~45 kDa; arrow). WT PLS3 purified under the same conditions is shown for comparison. **e** DSF melting curves of the T578NfsX4 and A589QfsX21 constructs (shown in **d**) are very similar and display two peaks: 1 – corresponds to the full-length construct (albeit significantly destabilized compared to the WT PLS3); 2 – coincides with the melting peak of the purified recombinant RD-ABD1 construct.

Supplementary Figure S2

a

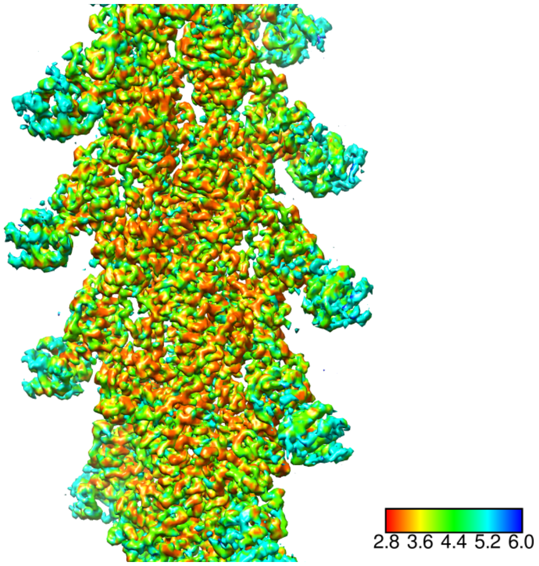

b

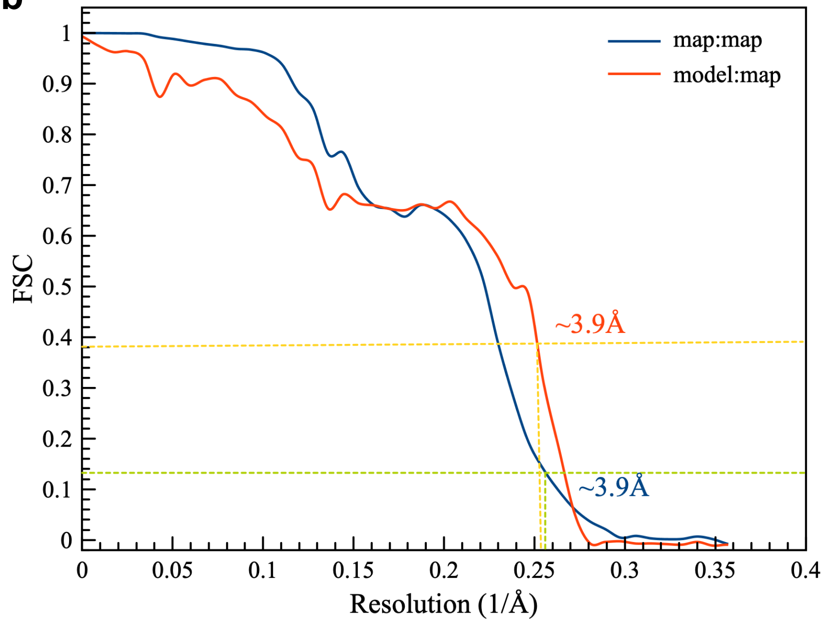

Supplementary Figure S2. The resolution estimation of the cryo-EM reconstruction of ABD2 PLS2 decorated F-actin (see also **Fig. 2a**). **a** Local resolution estimation shows the resolution is progressively getting lower at the distal regions of ABD2. **b** The overall resolution was calculated by two types of methods, map:map and model:map. The blue curve shows FSC between two-independent half maps, a resolulution of 3.9 Å at FSC=0.143. The red curve shows model:map FSC, which is also 3.9 Å at FSC=0.38.

Supplementary Figure S3

```
                                                                                   ααα αααααααααα                    α
           PLS1_ABD2  387  .......... .......... .......... .......... .......DMN LLEGES.KEE RTFRNWMNS. ..LGVNPYIN HLYSDLADAL  425
           PLS2_ABD2  386  .......... .......... .......... .......... .......DWG ALEGET.REE RTFRNWMNS. ..LGVNPRVN HLYSDLSDAL  424
           PLS3_ABD2  389  .......... .......... .......... .......... .......DWT LLEGET.REE RTFRNWMNS. ..LGVNPHVN HLYADLQDAL  427
       S.p.Fim1_ABD2  375  .......... .......... .......... .......... ......PEIE PFDAEGEREA RVFTLWLNS. ..LDVTPSIH DFFNNLRDGL  415
       A.t.Fim1_ABD2  382  .......... .......... .......... .......... ......AEMM TEDVETCRDE RCYRLWINS. ..LGIDSYVN NVFEDVRNGW  422
         FilaminA_ABD    1  .......... ..MSSSHSRA GQSAAGAAPG GGVDTRDAEM PATEKDL..A EDAPWKKIQQ NTFTRWCNEH LKCVSKR.IA NLQTDLSDGL   75
        α-actinin1_ABD    1  .......... ....MDHYDS QQTNDYMQPE EDWDRDLLLD PAWEKQ.... ........QR KTFTAWCNSH LRKAGTQ.IE NIEEDFRDGL   63
    β-III-spectrin_ABD    1  MSSTLSPTDF DSLEIQGQYS DINNRWDLPD SDWDNDSSSA RLFERSRIKA LADEREAVQK KTFTKWVNSH LARVTCR.VG DLYSDLRDGR   89
        Dystrophin_ABD    1  .......... .......... .......... .......... MLWWEEV..E DCYEREDVQK KTFTKWVNAQ FSKFGKQHIE NLFSDLQDGR   48
         Utrophin_ABD    1  .......... .......... ......MAKY GEHEASPDNG QNEFSDIIKS RSDEHNDVQK KTFTKWINAR FSKSGKPPIN DMFTDLKDGR   64
            Consensus       .......... .......... .......... .......... .......... ........#. .t%t.W.Ns. ........!. .l..#lr#gl

                           ααααααααα                        αααααααααα αααααα                            ααα αααααααααα ααα  ααααα
           PLS1_ABD2  426  VIFQLYEMIR V.PVNWSHVN KPPYPA.LGG NMKKIENCNY AVELGKNKAK FSLVGIAGQD LNEGNSTLTL ALVWQLMRRY TLN..VLSDL  511
           PLS2_ABD2  425  VIFQLYEKIK V.PVDWNRVN KPPYPK.LGG NMKKLENCNY AVELGKNQAK FSLVGIGGQD LNEGNRTLTL ALIWQLMRRY TLN..ILEEI  510
           PLS3_ABD2  428  VILQLYERIK V.PVDWSKVN KPPYPK.LGA NMKKLENCNY AVELGKHPAK FSLVGIGGQD LNDGNQTLTL ALVWQLMRRY TLN..VLEDL  513
       S.p.Fim1_ABD2  416  ILLQAYDKIT PNTVNWKKVN KAPASGDEMM RFKAVENCNY AVDLGKNQ.G FSLVGIQGAD ITDGSRTLTL ALVWQMMRMN ITK..TLHSL  502
       A.t.Fim1_ABD2  423  ILLEVLDKVS PSSVNWKHAS KPPIK....M PFRKVENCNQ VIKIGKQL.K FSLVNVAGND IVQGNKKLIL GLLWQLMRFH MLQ..LLKSL  505
         FilaminA_ABD   76  RLIALLEVLS QKKMHRKHNQ RPT......F RQMQLENVSV ALEFLDRE.S IKLVSIDSKA IVDGNLKLIL GLIWTLILHY SISMPMWDEE  158
        α-actinin1_ABD   64  KLMLLLEVIS GERLAKP..E RGK......M RVHKISNVNK ALDFIASK.G VKLVSIGAEE IVDGNVKMTL GMIWTIILRF AIQDISVE..  142
    β-III-spectrin_ABD   90  NLLRLLEVLS GEILPKP..T KGR......M RIHCLENVDK ALQFLKEQ.K VHLENMGSHD IVDGNHRLTL GLVWTIILRF QIQDISVETE  170
        Dystrophin_ABD   49  RLLDLLEGLT GQKLPK...E KGS......T RVHALNNVNK ALRVLQNN.N VDLVNIGSTD IVDGNHKLTL GLIWNIILHW QVKNVMKNIM  128
         Utrophin_ABD   65  KLLDLLEGLT GTSLPK...E RGS......T RVHALNNVNR VLQVLHQN.N VELVNIGGTD IVDGNHKLTL GLLWSIILHW QVKDVMKDVM  144
            Consensus       .ll.ll#..s .......... k........m r...leNvn. al...k.... ..Lv.i.g.d iv#Gn.k$tL g$.W..il.. ..........

                                      αααααααααα αααα                         ααααααααα αα              αα αααααααααα ααααα α
           PLS1_ABD2  512  GE...GEKVN DEIIIKWVNQ TLKSANKKTS ISSFKDKSIS TSLPVLDLID AIAPNAVRQE MIRRENLSDE DKLNNAKYAI SVARK.IGAR  597
           PLS2_ABD2  511  GG...GQKVN DDIIVNWVNE TLREAKKSSS ISSFKDPKIS TSLPVLDLID AIQPGSINYD LLKTENLNDD EKLNNAKYAI SMARK.IGAR  596
           PLS3_ABD2  514  GD...GQKAN DDIIVNWVNR TLSEAGKSTS IQSFKDKTIS SSLAVVDLID AIQPGCINYD LVKSGNLTED DKHNNAKYAV SMARR.IGAR  599
       S.p.Fim1_ABD2  503  SRG..GKTLS DSDMVAWANS MAAKGGKGSQ IRSFRDPSIS TGVFVLDVLH GIKSEYVDYN LVTDGS.TEE LAIQNARLAI SIARK.LGAV  588
       A.t.Fim1_ABD2  506  RSRTLGKEMT DADILSWANR KVRTMGRKLQ IESFKDKSLS SGLFFLNLLW AVEPRVVNWN LVTKGE.TDD EKRLNATYIV SVARK.LGCS  593
         FilaminA_ABD  159  EDEEAKKQTP KQRLLGWIQN KL....PQLP ITNF.SRDWQ SGRALGALVD SCAPGLCPDW DSWD....AS KPVTNAREAM QQADDWLGIP  239
        α-actinin1_ABD  143  ......ETSA KEGLLLWCQR KTAPY.KNVN IQNF.HISWK DGLGFCALIH RHRPELIDYG KLR.....KD DPLTNLNTAF DVAEKYLDIP  219
    β-III-spectrin_ABD  171  DNKE..KKSA KDALLLWCQM KTAGY.PNVN VHNF.TTSWR DGLAFNAIVH KHRPDLLDFE SLK.....KC NAHYNLQNAF NLAEKELGLT  250
        Dystrophin_ABD  129  AGLQ..QTNS EKILLSWVRQ STRNY.PQVN VINF.TTSWS DGLALNALIH SHRPDLFDWN SVVC....QQ SATQRLEHAF NIARYQLGIE  210
         Utrophin_ABD  145  SDLQ..QTNS EKILLSWVRQ TTRPY.SQVN VLNF.TTSWT DGLAFNAVLH RHKPDLFSWD KVV.....KM SPIERLEHAF SKAQTYLGIE  225
            Consensus       ......k... ...ll.W... k......... !.nF...sws .gl...al.h ...p.l.... .v........ ....na..a. s.Ark.lg..

                                           ααααα ααααααα
           PLS1_ABD2  598  IYALPDDLVE VK...PKMVM TVFACLMGKG LNRIK..     629
           PLS2_ABD2  597  VYALPEDLVE VN...PKMVM TVFACLMGKG MKRV...     627
           PLS3_ABD2  600  VYALPEDLVE VK...PKMVM TVFACLMGRG MKRV...     630
       S.p.Fim1_ABD2  589  IFILPEDIVA VR...PRLVL HFIGSLMAV. .......     614
       A.t.Fim1_ABD2  594  VFLLPEDIVE VN...QKMIL ILTASIMYWS LQR....     623
         FilaminA_ABD  240  QVITPEEIVD PNV.DEHSVM TYLSQFPKAK LKPGAPL     675
        α-actinin1_ABD  220  KMLDAEDIVG TARPDEKAIM TYVSSFYHAF SGAQKAE     256
    β-III-spectrin_ABD  251  KLLDPED.VN VDQPDEKSII TYVATYYHYF SKMKALA     287
        Dystrophin_ABD  211  KLLDPED.VD TTYPDKKSIL MYITSLFQVL PQQVSIE     246
         Utrophin_ABD  226  KLLDPED.VA VQLPDKKSII MYLTSLFEVL PQQVTID     261
            Consensus       ..l.pE#iV. v...d.k.!. ty..s..... .......
```

Supplementary Figure S3. Multiple sequence alignment of ABD2 domains of human plastin isoforms and ABDs of various t-CH-domain proteins. Multiple sequence alignment was performed using MultAllin (http://multalin.toulouse.inra.fr). Critical actin-binding residues identified in a recent cryo-EM structure of filamin A (PDB ID: 6D8C) are highlighted in yellow (nonconserved) and red (conserved between filamin and ABD2 of human plastins); corresponding conserved plastin residues are in magenta (see also **Fig. 2f**). Residues of the L478P-harboring loop are underlined. Residues of the hydrophobic cluster formed by the 478 loop (shown in **Fig. 2e**) are highlighted in cyan. The secondary structure is denoted based on PDB ID: 6VEC. The consensus sequence indicates sequence conservation: uppercase, >90% ; lowercase, 50%-90%; !, (I or V); $, (L or M); %, (F or Y); #, (N, D, Q, or E).

Supplementary Figure S4

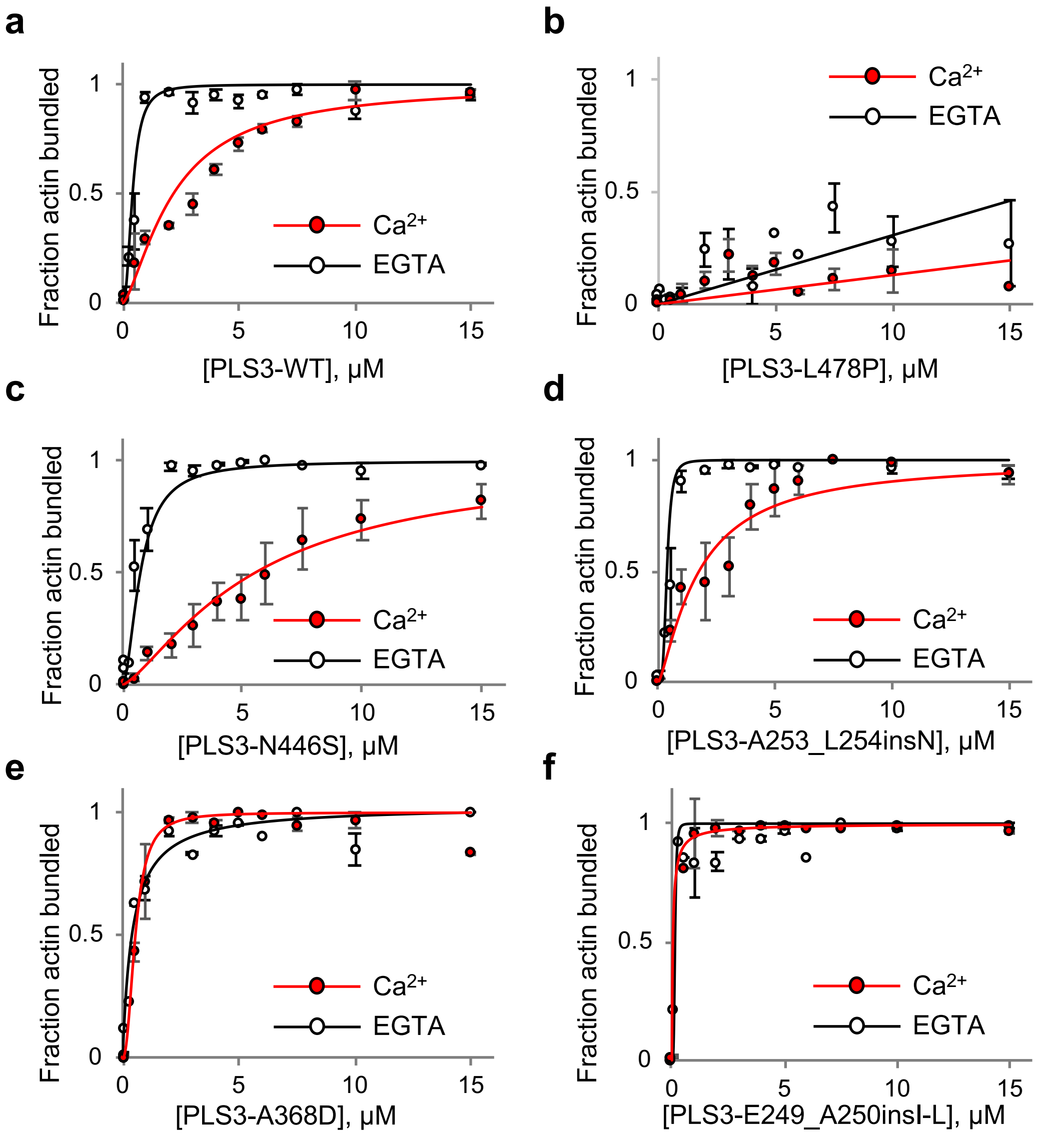

Supplementary Figure S4. F-actin bundling by WT and mutated PLS3. **a-f** F-actin bundling by WT and mutated PLS3 was analyzed by low-speed co-sedimentation in the presence (Ca^{2+}) or absence (EGTA) of calcium. Note that the bundling data in the presence of EGTA are the same as shown on **Fig. 1e**. $[PLS3]_{50\%}$ are given in Table 1. Error bars represent standard errors of the mean.

Supplementary Figure S5

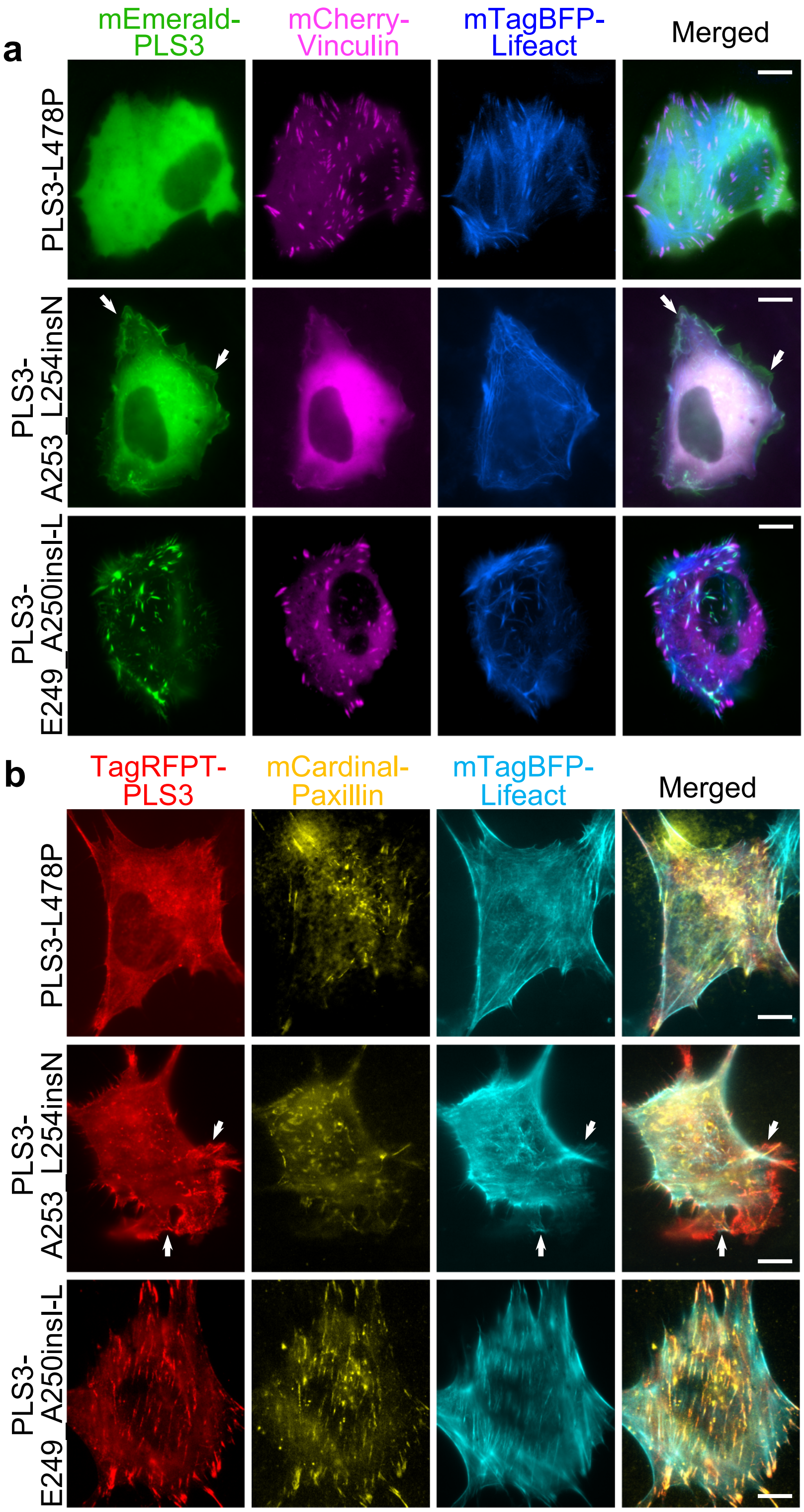

Supplementary Figure S5. Effects of OI-linked PLS3 mutations on cellular localization of PLS3 in osteoblasts and osteocytes (see also **Fig. 3a,b**). **a** Live-cell TIRFM imaging of U2OS osteoblasts co-transfected with mEmerald-PLS3 constructs (green), mCherry-Vinculin (FA, magenta) and mTagBFP-Lifeact (F-actin, blue). **b** Live-cell TIRFM imaging of Ocy454 osteocytes co-transfected with TagRFPT-PLS3 constructs (red), mCardinal-Paxillin (FA, yellow) and mTagBFP-Lifeact (F-actin, cyan). White arrows in **a** and **b** point at membrane ruffles; scale bars are 10 μm.

Supplementary Figure S6

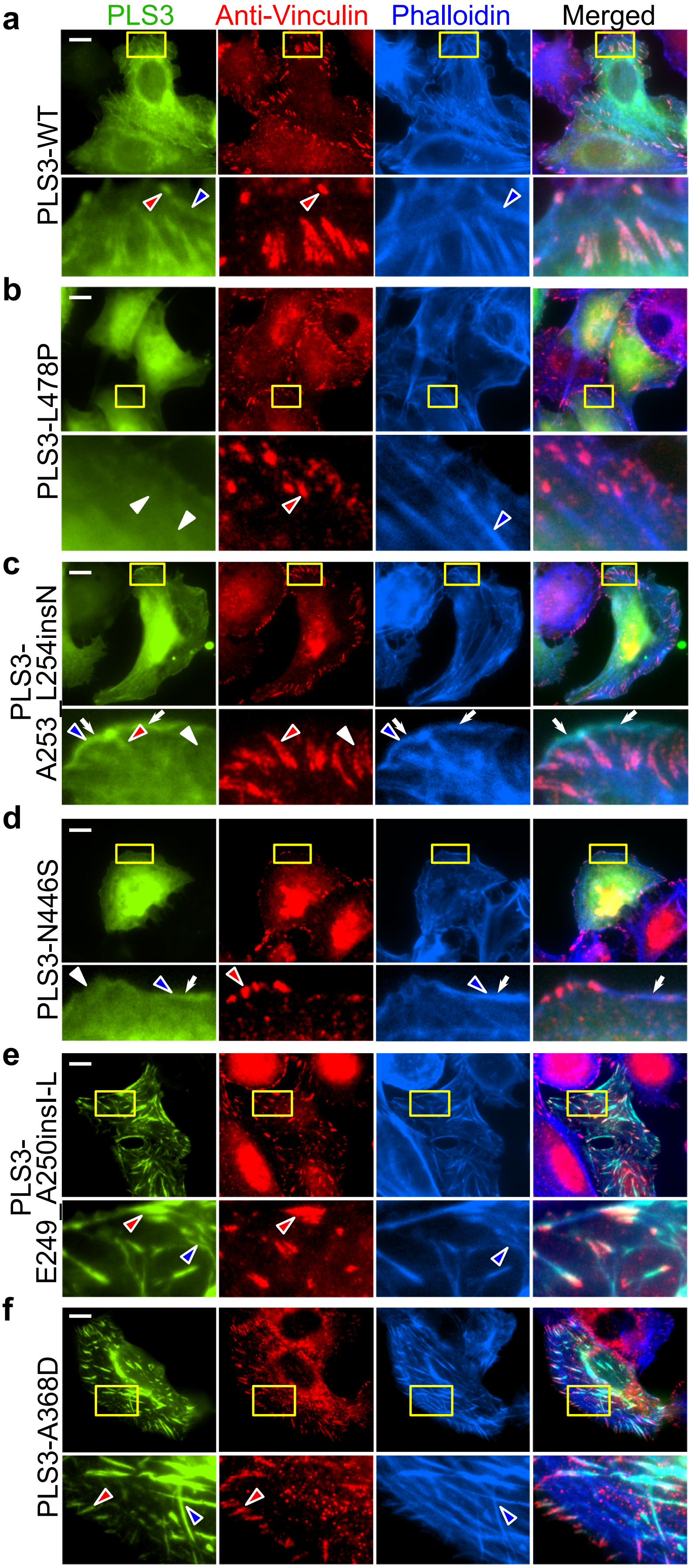

Supplementary Figure S6. Effects of OI-linked PLS3 mutations on cellular localization of PLS3 and morphology of focal adhesions in U2OS osteoblasts (see also **Fig. 3c-e**). **a-f** Cellular localization of PLS3 variants was assessed in fixed U2OS cells transfected with mEmerald-PLS3 constructs (green). Endogenous FAs were detected by immunostaining with anti-vinculin antibody (red); SFs were revealed by co-staining with coumarin-phalloidin (blue). Enlarged view of the boxed areas are given below each image. Blue arrowheads exemplify co-localization of PLS3 with F-actin bundles; red and white arrowheads point at FAs enriched and depleted by PLS3, respectively; white arrows point at membrane ruffles. Scale bars are 10 μm.

Supplementary Figure S7

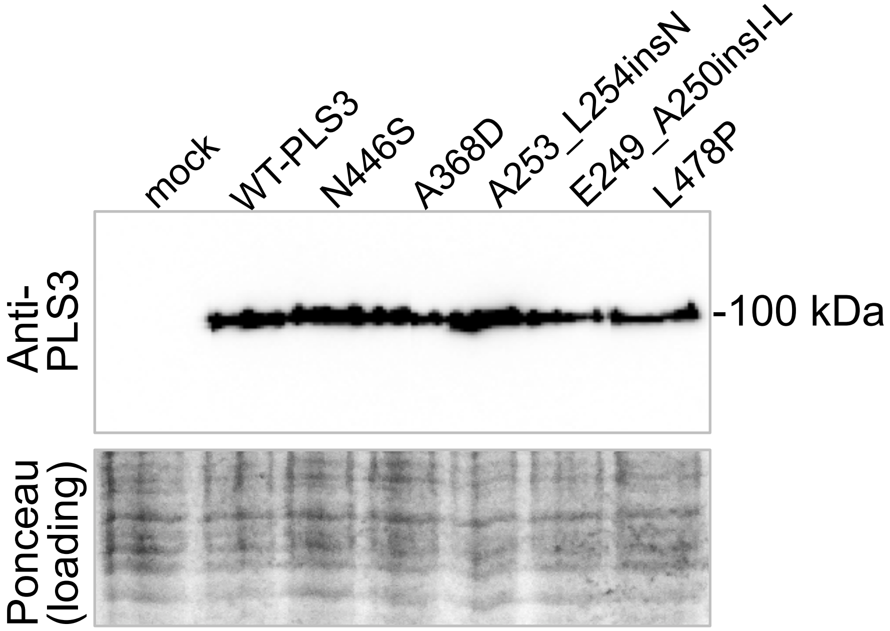

Supplementary Figure S7. Expression of mEmerald-tagged PLS3 constructs in U2OS cells upon transfection (see also **Supplementary Fig. S6**). Expression of mEmerald-tagged PLS3 constructs (99 kDa) was analyzed by anti-PLS3 western blotting. Ponceau S staining is shown as a loading control; *mock*, mock-transfected cells to demonstrate the signal specificity.

Supplementary Table S1. Cryo-EM data collection, reconstruction, and model refinement statistics

**Cryo-EM Data Collection**	
Microscope	Titan Krios
Voltage (kV)	300
Camera	Falcon III
Pixel size (Å)	1.4
Defocus range (μm)	-1.5 ~ -2.5
**Helical Reconstruction**	
Number of movies	3,135
Number of segments	248,184
Map Resolution (Å)	3.9
Map sharpening B-factor ($Å^2$)	-193
**Model Refinement and Validation**	
MolProbity score	1.63
Clashscore	3.68
Poor rotomers (%)	0.95
**Ramachandran Plot**	
Favored (%)	92.33
Allowed (%)	7.17
Outliers (%)	0.49
RMSD bonds (Å)	0.008
RMSD bond angles (°)	1.028

Supplementary Table S2. Distances in the hydrophobic cluster formed by the loop bearing OI-associated PLS3 mutation L478P (L475 in PLS2 numbering)

				Distance (Å) between L475 of PLS2 (or its equivalent) and a corresponding residue in the hydrophobic cluster			
	**PDB ID**	**Resolution (Å)**	**L475** (equivalent)	**V464** (equivalent)	**F473** (equivalent)	**L494** (equivalent)	**I478** (equivalent)
ABD2 PLS2	*6VEC*	*3.9*	*L475*	*4.713*	*4.108*	*3.907*	*3.874*
filamin A	6D8C	2.3	(L121)	3.890 (L111)	4.476 (I119)	3.981 (L140)	3.937 (I124)
S. pombe fimbrin	1RT8	2	(L467)	4.235 (V457)	3.683 (F465)	3.503 (L486)	4.438 (I470)
utrophin	1QAG	3	(L107)	4.541 (L97)	4.541 (V105)	4.751 (L126)	3.894 (I110)
dystrophin	1DXX	2.6	(L91)	3.968 (L81)	4.164 (V89)	3.622 (L110)	4.568 (I94)
α-actinin 1	1TJT	1.7	(L121)	4.248 (L111)	4.317 (V119)	5.327 (M140)	4.074 (I124)
